# Experiences of Transforming a Complex Nephrologic Care and Research Database into i2b2 Using the IDRT Tools

**DOI:** 10.1155/2019/5640685

**Published:** 2019-01-17

**Authors:** Christian Maier, Jan Christoph, Danilo Schmidt, Thomas Ganslandt, H. U. Prokosch, Stefan Kraus, Martin Sedlmayr

**Affiliations:** ^1^Chair of Medical Informatics, Friedrich-Alexander-Universität Erlangen-Nürnberg, Erlangen, Germany; ^2^Medizinische Klinik für Nephrologie, Charité–Universitätsmedizin Berlin, 10117 Berlin, Germany

## Abstract

The secondary use of data from electronic medical records has become an important factor to determine and to identify various causes of disease. For this reason, applications like informatics for integrating biology and the bedside (i2b2) offer a GUI-based front end to select patient cohorts. To make use of those tools, however, clinical data need to be extracted from the Electronic Health Record (EHR) system and integrated into the data schema of i2b2. We used TBase, a documentation system for nephrologic transplantations, as a source system and applied the Integrated Data Repository Toolkit (IDRT) for the Extract, Transform, and Load (ETL) process to load the data into i2b2. Since i2b2 uses an entity-attribute-value (EAV) schema, which is a fundamentally different way of modeling data in comparison to a standard relational schema in TBase, we evaluated if (a) the data relationship of the source system entities can still be represented in the i2b2 schema and if (b) the IDRT is a suitable solution for loading the data of a comprehensive data schema like TBase into i2b2. For that reason, we identified entities in the TBase data schema which were relevant for answering questions on cohort identification. By doing so, we found out that the entities had different structures that needed to be handled differently for the ETL process. Furthermore, the use of IDRT revealed shortcomings with regard to large input data and specific data structures that are part of most modern EHR systems. However, this project also showed that our way of modeling the TBase data in i2b2 has been proven to be successful in terms of answering the most common questions of clinicians on cohort identification.

## 1. Introduction

The secondary use of data from electronic medical records has become an important factor to determine and to identify various causes of disease as well as to figure out what kind of therapy would be the most effective [1]. Patient-centric documentation systems, such as electronic patient records, usually do not provide functions to select patient cohorts, although this is a major requirement for the secondary use of clinical data [2]. Clinicians rarely have the skills to work directly on databases; this barrier makes database experts necessary, who are able to select patient cohorts by means of SQL queries and to make the aggregated data available to the clinicians. Yet, there are systems that enable the clinician to explore the data on his own, especially in secondary use. A well-known application for this purpose is informatics for integrating biology and the bedside (i2b2) [3], which provides comprehensive graphical user interface- (GUI-) based functions, “allows ad-hoc queries to be created by research clinicians themselves […] and returns aggregate numbers of patients that satisfy the queries” [3].

Within the scope of the project “Clinical Data Intelligence,” we defined a use case which comprised the integration of clinical data from TBase [4, 5], a documentation system for nephrology with focus on transplantations, into i2b2. Our experience with i2b2 [6, 7] and its flexible data schema in previous projects motivated us to choose i2b2 as a research database. Thus, a database with a common data model should be built, which allows access for the other project partners. On the part of the project partners, different modules should be developed that analyze the data and write new knowledge back to the research database. In the end, the research database should comprise data from different sources that is integrated with i2b2 and allows for an easy access of the clinicians through the i2b2 front end.

i2b2 enables clinicians to explore clinical data on their own and without requiring SQL or programming skills. It comes with its own database and a proprietary data schema. In contrast to clinical electronic record systems that normally use proprietary relational database schemas, i2b2 makes use of an entity-attribute-value (EAV) schema. This is a widely used technique for clinical data repositories [8], as it provides a generic data warehouse like star schema [9] (i.e., a fact table and several dimension tables) which allows straightforward integration of heterogeneous data. It thus reduces the complexity of mapping diverse relational schemas to a common data model and allows for the comparison of data of different sources and formats. In order to provide the clinical data in i2b2, there is a need for an Extract, Transform, and Load (ETL) process which allows the integration of data gained from TBase records into the i2b2 data schema. A literature research yielded some already published projects [10, 11] which provide approaches to the integration of data into i2b2. However, these approaches expect the source data to be present in a specific format so that they are normally not transferable to different source data schemas. Instead of making yet another proprietary approach or using an integration tool that requires a specific source data format, we decided to evaluate the use of the Integrated Data Repository Toolkit (IDRT), which promises to be a “platform for easy integration and administration of data from heterogeneous sources” [12] and offers the possibility to provide a configuration with which the schema of the source data can be defined.

The process of transforming data from one schema to another may be accompanied by a loss of coherence between the entities of the source data schema (i.e., a loss of information). In our case, both schemas represent fundamentally different ways of modeling data, which may result in the situation that relationships of the source data schema cannot be maintained in the target schema due to the model-specific way of saving a relationship. Therefore, we were interested in whether an ETL process from TBase into the i2b2 data schema is accompanied by a loss of coherence and if so, to which extent specific data can be mapped without losing any relationships between the entities. To determine the quality of the mapping, we evaluated (a) whether the clinicians' typical questions on cohort identification could still be answered at all and if so, whether they could be answered, (b) in the graphical front end of i2b2, or (c) only by the help of SQL on the i2b2 database itself. Furthermore, we wanted to find out which kind of questions could not be answered anymore because of the aforementioned possible loss of coherence due to the limitations of the target data schema or to the range of functions in the IDRT.

## 2. Materials and Methods

The first step was to look for publications about i2b2 in a nephrologic context by using search expressions like “i2b2 nephro∗” or “i2b2 transplantation” in PubMed, but this did not yield a result. After that, we examined the data schema of TBase together with clinical domain experts to sort out entities that are generally relevant to the purpose of identifying patient cohorts. The next step was to analyze the data structure of these entities and to examine potential differences between them. Concurrently, we conducted a literature research and compared integration tools for i2b2. Then a preprocessing of the source data schema prepared every type of data structure in order to pass it on to the integration tool. After loading the data, the mapping was evaluated by trying to answer clinicians' formerly stated research questions on cohort identification in i2b2.

### 2.1. About TBase

TBase [4, 5] is a web-based electronic patient record that focuses on clinical documentation in the context of kidney transplantations and is currently used in eight German university hospitals. Data which are documented in TBase are comprehensive: TBase comprises data on patient history and drug therapy data, as well as laboratory results and it fully supports the complete pre- and posttransplantation documentation as well as the documentation of follow-up data. Since all organ recipients are chronically ill, a huge amount of data arise over their lifetime. Consequently, TBase has to cope with extensive data volumes.

### 2.2. Integration Tool for i2b2

In our literature research, we focused on integration tools that do not require a specific source data structure in order to follow a generic approach that can be transferred to other source data models as well. Having compared different tools [10, 11, 13, 14], we decided to use the “Integrated Data Repository Toolkit” (IDRT) [12], which is an open source software and has been created with Talend Open Studio for data integration [15]. It performs a transform and load of “various formats like CSV, SQL, CDISC, ODM, or biobanks” [12] which addresses the i2b2 schema and which includes a GUI-supported ontology editor for rearranging and mapping patient data.

### 2.3. Preprocessing and ETL

Together with clinical domain experts, we identified entities of the TBase data schema which are relevant for answering the typical questions on the identification of patient cohorts. Then the data schema of TBase was examined to identify potentially different data structures among the relevant entities. Each variant needed to be handled differently to prepare the data for the loading process: some entities, for example, were lacking attributes that are mandatory for representing a fact in i2b2. As a consequence, parts of the data schema were denormalized to fulfill the requirements. Lastly, the IDRT carried out the transformation and load process.

### 2.4. Evaluation

Eventually, we determined possible error messages of the IDRT during the loading procedure and performed a comparison of counts of facts for each entity on both databases. Furthermore, we evaluated whether our way of mapping and transforming the TBase source data and their integration into the i2b2 schema still allows for the answering of the typical questions of clinicians on the identification of cohorts. The TBase experts who were responsible for processing the clinicians' requests for cohort identification provided us with four typical questions which the clinicians had posed. Afterwards, we decomposed the questions according to data-specific criteria and determined which question could or could not be answered anymore. We examined whether the questions could be answered within the graphical front end of i2b2, whether they could be answered only by the help of SQL on the i2b2 database itself, or if they could not be answered at all because of a missing relationship. The latter might have been caused by the fact that i2b2 was not able to maintain the relationship because of its generic data warehouse format or by the fact that IDRT was lacking a feature to load that kind of data.

## 3. Results

### 3.1. Source Data Quantification

The source dataset covers 18 years of medical documentation and comprised data of 3,493 patients who, all in all, had 3,767 kidney transplants, 54,009 diagnoses, 182,250 medications, and 8,652,557 laboratory values.

### 3.2. Examining the Source Data

**Figure 1:**
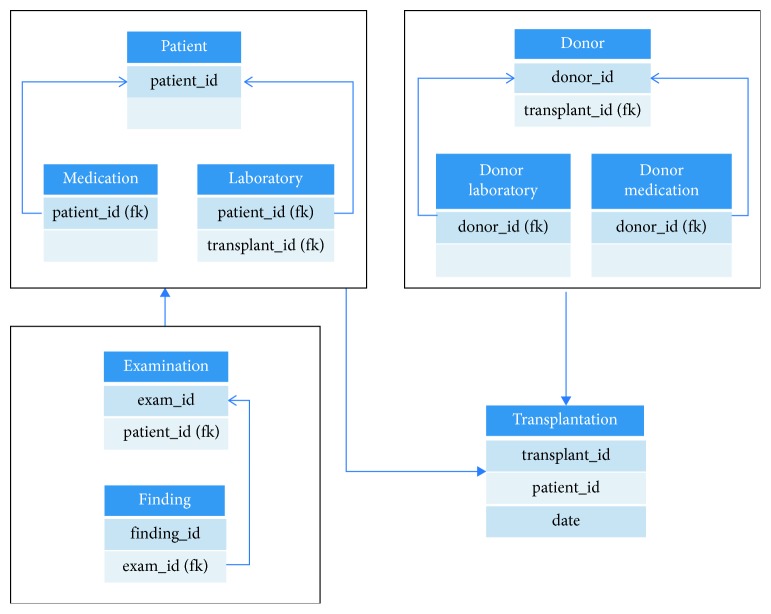
Simplified excerpt of the TBase data schema illustrating data coherences.

Generally, the patient is the central entity of clinical documentation which means that documented information is directly referred to him. After examining the data schema of TBase, however, we found out that the transplantation is the central entity in the data schema: Figure 1 shows a simplified excerpt of the TBase data schema which illustrates that the documentation does not only refer to the patient. Of course, there are data items such as findings or examinations that refer to the patient. In the context of nephrologic transplantations, however, there is also data on the entity of the organ donor. Both datasets (patient and donor) are linked through the transplantation entity, which turned out to be the central entity in the use case of a nephrologic transplantation.

### 3.3. Analyzing the Source Data

Together with domain experts, we identified entities of the TBase database schema, which they classified as relevant to the typical questions of clinicians on cohort identification. The database tables included several foreign key constraints, which could not be directly mapped to the i2b2 EAV schema because of the data coherence in i2b2. i2b2 offers the possibility to represent relations within the dataset by a three-level concept that includes the patient as a first level, the encounter as a second level, and the use of modifiers [16] as an optional third level.

To represent a so-called “fact” (i.e., an individual clinical data record), the i2b2 EAV schema requires several attributes. These attributes include at least the encounter number, the patient identifier, the date of when the fact occurred, and the fact itself. In order to prepare the data for the ETL process, we identified potentially different structures among all tables and the items which needed to be joined to prepare every kind of table with the aforementioned attributes. Thus, we analyzed the entities and determined five types of tables, which needed to be handled differently in terms of ETL preprocessing. In doing so, these five types are separated again in two different categories. On the one hand, the data structure of the entities needs to be considered and on the other hand the data itself:

- Data structure
  1. Tables which are already present in an i2b2-compliant EAV schema
  2. Tables which are not present in an i2b2-compliant EAV schema and which comprise a foreign key which references the transplantation table or another table including a foreign key to a table which references the transplantation table
  3. Tables which are not present in an i2b2-compliant EAV schema and which do not comprise a foreign key which references the transplantation table or any other key that would enable a reference to the transplantation table.
- Data
  (4) Tables which comprise free-text blobs: there is also a need to look at the data itself as i2b2 defines special data schema structures for specific types of data. This is particularly the case when there are free-texts, such as pathology findings.
  (5) Tables which comprise laboratory values with associated units

**Figure 2:**
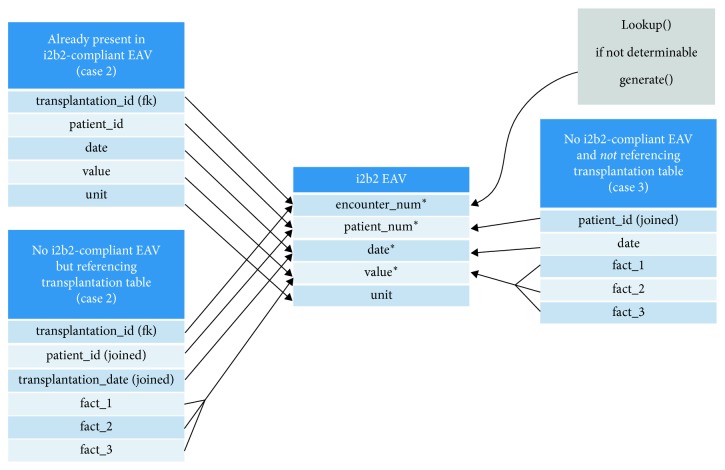
Simplified illustration of determined table types (1 to 3) mapped to i2b2 EAV.

All five types are present in TBase so that there was the need to take them into account and to handle them separately.  Figure 2 illustrates the mapping of tables that refer to data structure issues (cf. i., types 1 to 3).

### 3.4. Preprocessing of the Source Data

Generally, in a normalized relational data schema—as it is the case with the TBase schema—every type of clinical fact is located in a separate database table, which might not necessarily comprise the aforementioned mandatory attributes. Therefore, there is a need to equip every single table with the aforementioned columns to prepare them for the loading procedure.

As mentioned above, an encounter number is one of the mandatory attributes in i2b2. However, the clinical case number in the TBase database, which would be equivalent to the encounter number, is not present in all tables and cannot be joined to every entity because of the missing relationships. We found that the main entity of a nephrologic data schema in the context of transplantation is the transplantation table, which is related to most of the other database tables by a foreign key constraint. As a consequence, the transplantation identifier was used as the encounter number for the i2b2 schema instead of the original clinical case number. With that solution, all data related to a transplantation (i.e. has a foreign key constraint to the transplantation table) can be bound to this identifier and do not exceed the number of concept levels offered by i2b2 (cf.  Section 3.3).

Thus, the preprocessing comprised the creation of a database view which joined the mandatory attributes of the transplantation table (cf. Figure 1) to every table in which these were originally missing. However, this is only necessary if the table does not comprise all mandatory attributes. Moreover, this is only possible if the respective table comprises a foreign key which references the transplantation table or another table which includes a foreign key to a table that references the transplantation table.

#### 3.4.1. Preparing the Tables of Type 1

The IDRT is not suitable for processing tables that are already present in an i2b2-compliant EAV schema out of the box. This means that all mandatory attributes needed for representing a fact in i2b2 (cf.  Section 3.3) are either part of a data row of a source database table or created in a separate preprocessing step (cf. Preprocessing of the source data). If these data rows were rotated, the relation between the attributes in i2b2 would get lost, which would result in an unusable representation of these facts in the i2b2 front end. The architecture of the processing jobs in the IDRT is modularized so that we adapted it for that use case (cf. Adapting the IDRT below).

#### 3.4.2. Preparing the Tables of Type 2

The tables of type 2 could be loaded into i2b2 by the standard implementation of the IDRT as all necessary attributes could be joined to the transplantation table by foreign keys.

#### 3.4.3. Preparing the Tables of Type 3

The tables of type 3 do not comprise a reference to the transplantation table so that it would not be possible to use the transplantation identifier as the encounter number in i2b2. In this particular case, we tried to map the clinical fact by the patient id as well as a reference to the date on which the fact has been collected, which is generally available in every table of this kind. To this end, we defined a timeframe that starts two weeks before the transplantation and ends two weeks after a possible organ failure. If the organ does not fail, all clinical facts which will be collected later on will be referenced to the given transplantation as long as there is no further transplantation. For all other clinical facts that do not fit into any timeframe, a surrogate encounter number was generated so that there will not be any loss of information. The extension of the timeframe to two weeks before and after the transplantation has been recommended by the clinicians as some fact data may have been entered shortly before a transplantation or after an organ failure.

We used this strategy, for example, for the examination table (cf. Figure 1). It does not comprise a reference to a transplantation, but it took place because a transplantation was imminent or had already happened. As i2b2 requires an encounter number, we tried to reference every fact to a transplantation although it might not have been referenced in the source data. If the date of examination did not occur within the aforesaid timeframe of two weeks, a surrogate encounter number was generated.

After the mandatory attributes were joined to the tables, the loading could be performed by the standard implementation of IDRT.

#### 3.4.4. Preparing the Tables of Types 4 and 5

A proprietary approach was needed to load free texts, such as pathology findings or units of laboratory values. As the IDRT does not support the loading of these kinds of data out of the box, we adapted its pipeline so that it also accepts free text blobs as input format and reported this extension back to the developers.

Laboratory findings are usually present in diverse units so that it is important to have a reference to the respective unit as its value could not be interpreted otherwise.

### 3.5. Modifications to the IDRT

We modified the IDRT to allow the loading of data according to tables of type 1 (tables already being present in an i2b2-compliant EAV schema), type 4 (free-text blobs), and type 5 (laboratory values with associated units).

#### 3.5.1. Modifications for Type 1

**Figure 3:**
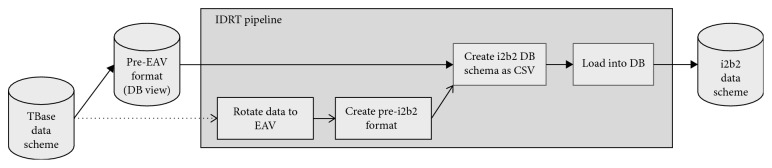
IDRT pipeline with disabled subjobs.

Figure 3 shows the IDRT pipeline and how it is structured into subjobs. Originally, after retrieving the source data, it is rotated to EAV, brought to a proprietary IDRT pre-EAV format, transformed to the proper i2b2 schema, and finally loaded into the database. To skip the data rotation, however, we disabled the call of both subjobs, which are responsible for the data rotation and the creation of the pre-EAV format (grayed out in Figure 3) and all actions in between. After reading the input file, the pipeline directly continues with the transformation from the pre-EAV to the proper i2b2 format. This requires the source data to be pretransformed to the pre-EAV format. This is done in the source database by the creation of a database view. The schema of this view exactly corresponds to the pre-EAV format of IDRT so that it can be read into the IDRT pipeline (with disabled subjobs) straightforwardly (cf. Figure 3).

#### 3.5.2. Modifications for Type 4

Originally, the IDRT did not support the loading of free-text blobs. We adapted it to accept free-text values and fill them into the OBSERVATION_BLOB column in the OBSERVATION_FACT table.

#### 3.5.3. Modifications for Type 5

Originally, the IDRT did not support the loading of laboratory values with associated units. We adapted it to also accept unit values beside the fact for a dataset. After the adaption, the IDRT was able to fill the UNITS_CD column in the OBSERVATION_FACT table.

### 3.6. Evaluation

To evaluate the data loading, we logged any error messages of the IDRT to be able to check if there are any problems during the loading procedure and performed a comparison of counts of facts for each entity on both databases. No error messages were reported, and the count of facts for each entity in both databases was equal.

To evaluate the mapping procedure, we received four questions on cohort identification of nephrologists. These questions had been answered by TBase experts on the TBase database by SQL in the past. Each question consisted of several inclusion and exclusion criteria. We identified the following categories, each of which represents a particular type of criterion:

1. All facts that had been collected at one event (i.e., one lab test, one visit)
2. Numerical values which are greater/smaller than or equal to a fixed value
3. Date values which are greater/smaller than or equal to a fixed value
4. The clinical event (fact) being NULL (i.e. value is not set)
5. The clinical event (fact) matches an exact string or not
6. A full-text string search using wildcards
7. The clinical events happened in temporal order with the
  - Boolean operators AND and OR as logical connectors
  - event A happening *x* days after the Event B

#### 3.6.1. Study 1

This study aims at patients for whom three specific types of lab values were collected in one and the same lab test.

In fact, i2b2 allows the grouping of datasets by specifying a so-called “instance num.” This identifier can be referenced in the i2b2 web client so that only selected facts that share the same instance num (i.e., collected at one and the same event) are returned. Consequently, this function of i2b2 would fulfill the requirements of criterion 1.

IDRT, however, does not allow the specification of custom instance nums and just uses an incremented value for this attribute. Therefore, it is not possible to fulfill criterion 1 with IDRT. The estimation to adapt the IDRT to offer a function that solves this issue would be comprehensive so that it could not be implemented within the scope of this project.

#### 3.6.2. Study 2

This study aims at patients who received a kidney transplant, who are still alive, whose HCV value (lab value) is positive and who had a follow-up after a given date X.

All requirements of the dedicated study criteria were fulfilled by both i2b2 and IDRT without any restrictions.

#### 3.6.3. Study 3

This study aims at patients who got a kidney transplant (but not together with a pancreas transplant) and whose kidney has been rejected and who suffered from a urinary tract infection between the transplantation and the rejection of the transplant.

Because i2b2 v1.7.00 supports temporal query constraints, it is possible to define a sequence of events which determine the order in which the events should have occurred. The fact that the patient suffered from a urinary tract infection is not available as a structured value so that the TBase experts had to use a wildcard to search for equivalent terms in free-texts. The i2b2 web client supports a free-text search with wildcards, so that there is no limitation of i2b2 in this respect.

IDRT, however, is originally not capable of loading free-text blobs into i2b2. Compared with the adaption in study 1, the complexity of the adaption of the IDRT was simpler in this case so that we implemented the corresponding function and also reported our changes to the developers.

#### 3.6.4. Study 4

This study aims at patients who suffer from a chronic kidney disease (CKD) stage and whose most recent collected hemoglobin value, which was collected 183 days after the transplantation at the earliest, is between two static values. Furthermore, they must not have had an erythropoiesis-stimulating agents (ESA) treatment for at least six months after the transplantation. The most recent urine albumin-to-creatinine ratio (UACR) must have been determined 183 days after the transplantation at the earliest and must not be higher than a given static value.

Since i2b2 v1.7.00, the web client supports the search of a fact/event that was collected/happened *X* days after another fact/event. So, there are no limitations on the part of the current version of i2b2.

There are no limitations on the part of IDRT.

Table 1 illustrates the criterion to study mapping described above and if there are issues that may occur with the use of IDRT or i2b2.

## 4. Discussion

### 4.1. Discussion of Methods

In order to select a suitable ETL tool, which enables a generic approach, we searched the literature for published work. Besides the IDRT, three other projects were taken into account, which could have been suitable approaches to our needs.

The ONCO-i2b2 platform [13] is an infrastructure that specializes in extracting data from the information system of the Fondazione Maugeri hospital, in integrating the data into the biobank, and loading the data into i2b2. Also, Haarbrandt et al. [11] provided a way to integrate data from openEHR-based data repositories into i2b2. Both solutions, however, require the source data to be available in a predefined format or call for a predefined hospital information system that provides the data. In comparison, IDRT allows for the creation of configuration files for input data in order to specify semantic information for attributes. This makes the IDRT suitable for any kind of input data.

The OntoSuite [14] is a flexible integration tool for i2b2 that accepts source data in any format by defining semantic coherences between the attributes of the input data. However, it only supports i2b2 1.6 and mainly focuses on complex semantic harmonization. The ETL process is defined as a declarative representation which is stored in resource description framework (RDF) triples, but which would have caused too much overhead for a straightforward integration of data.

Eureka! Clinical Analytics [10] is a loading tool that accepts an Excel sheet as an input and requires a predefined format for the input data, which makes it less flexible than the IDRT. Furthermore, the input data model is not substantial enough for using it with nephrologic data in the context of transplantation medicine, as it only covers patient data, medication data, and encounter data as well as ICD9 procedures, ICD9 diagnoses, and laboratory test results. Moreover, it is outdated as it only supports i2b2 1.5.

### 4.2. Discussion of Results

#### 4.2.1. IDRT

The IDRT promises to be a “platform for easy integration and administration of data” [5]. Indeed, the IDRT accepts generically structured input data, such as CSV, CDISC ODM, and SQL databases, and offers an easy configuration to define the required entities for the transformation procedure. Furthermore, the user does not even have to consider the i2b2 database schema since the IDRT acts as a black box, which means that it hides all the i2b2-specific ETL logic. Moreover, as stated in the publication of the IDRT, the handling is, indeed, relatively easy because of its GUI-based interface. At a first glance, these characteristics seem to make the IDRT a well-suited tool for our needs. However, there are some shortcomings that need to be examined further. When handling small input data (i.e., up to some megabytes), the IDRT pipeline works flawlessly and as expected. In our project, however, we are working with a source database that comprises database tables which can have a size of more than a gigabyte each. Due to the nature of the medical domain, there are lots of laboratory results for one patient collected before and after the transplantation as well as at every follow up. This lets the laboratory table grow quite fast and results in a size of 1.2 gigabytes after 18 years of medical history. So, when trying to load a file of that size, the IDRT consumes a lot of resources. To find a solution in order to limit this resource consumption, we had a look at the Talend Open Studio sources of the IDRT and found out some details:

1. The source data files are completely read into RAM and transformed into several interim formats, which are all written to disk in order to read them again when needed at a subsequent point of time. We suppose that either the Java-internal garbage collection does not remove these after writing them to disk or that there are still references remaining in the Java code, which prevent the interim results from being removed.
2. When the data are rotated to EAV, all datasets (one dataset is equivalent to one line in the source data file) are kept in RAM so that the amount of data grow rapidly as every dataset is transformed into *x*-*y* lines in the EAV model, whereby *x* corresponds to the number of columns in the source data and *y* to the number of meta-data columns (the start and/or the end date of an event for instance).

Both circumstances lead to a massive use of RAM, which grows rapidly with the rotation of the data to EAV and the progress of the pipeline process. There is no possibility to free resources manually in Java, except for the closing of connections of file readers or writers, so that one has to rely on the garbage collection.

As a workaround, most operating systems allow for the manual expansion of the swap space and therefore provide enough resources of swap space on disk, which will, however, result in poor performance when it comes to the use of magnetic disks. Yet, in this case, switching to a solid state drive can fasten the import significantly. Furthermore, the Java code is produced by the Talend Open Studio framework so that there is no possibility to influence the source code through potentially optimized resource management without losing the option of editing the jobs in Talend Open Studio.

Besides expanding the swap space, one could think of clustering big input files into several smaller ones to reduce the resources which are required for the transformation. The IDRT pipeline will perform a complete rerun for every file so that resources are cleaned after each run.

Apart from workarounds, a possible solution would have been the implementation of the IDRT with Talend Open Studio Extract-Load-Transform (ELT) components, “where the target database management system (DBMS) becomes the transformation engine” [17]. In this way, the resources on the client side can be saved and due to the DBMS optimizing queries itself, one can be sure that the execution of the transformation is achieved in a performant way.

Furthermore, we found out that, for every i2b2 database table (seven in total), one comma-separated file is generated. The format of each file corresponds to the associated table schema of the i2b2 database. In practice, however, it would be sufficient to only generate the format of the tables OBSERVATION_FACT, the I2B2, and PATIENT_DIMENSION table as the data of all the other necessary database tables can be generated by SQL queries. This approach would probably lead to a considerably lower consumption of resources on the client system and speed up the integration process.

The focus of the functions of the IDRT is on loading simple clinical facts like pure numerical or categorical values. The IDRT originally did not support the loading of special data types, such as free texts, data already present in EAV, or laboratory values and their units. These shortcomings were not mentioned in the aforesaid publication of the IDRT [12].

In terms of the laboratory values with associated units, it would have been possible to use modifiers to represent the units. However, this would have been a workaround that would not support unit conversion or the GUI-based functions for units. Consequently, we decided to adapt the IDRT tools to properly fill the UNITS_CD column in the OBSERVATION_FACT table.

### 4.3. Mapping Methodology

To avoid losing any relationships between the entities, the transplantation identifier of TBase was used as the encounter number in i2b2. In doing so, we lost the relationship to the original TBase encounter. However, the encounter numbers are generally used for billing purposes in Germany and are therefore not obligatory for secondary use in a medical context.

### 4.4. Evaluation of the Mapping

Our study serves as an example that it is possible to map even complex relational database schemas to the i2b2 star schema. Furthermore, i2b2 turned out to be a generic tool with regard to constructing queries on the i2b2 web client. All of our sample questions on cohort identification could be answered on the web client with the latest version of i2b2. However, i2b2 versions before 1.7.00 do not support temporal queries so that criterion 7b could not be answered on the web client in this case but on the database by the help of a SQL query [18]. This shows that the relationship between the TBase entities could be maintained in the i2b2 EAV model for the most recent questions of clinicians on cohort identification.

The IDRT is generic with respect to input data, as no predefined model is required. However, our studies showed that the IDRT does not support all kinds of data types/data models that are present in an electronic patient record system like TBase. Since the IDRT is open source, we were able to adapt it to our specific needs. There is quite some documentation of the IDRT available in the i2b2 Community Wiki; however, it only describes the use of the tools with the graphical user interface. We did not make use of the GUI and focused on the Talend Open Studio sources due to the adaptions of the IDRT (cf. Modifications to the IDRT). That would also involve changes of the source of the GUI to make the tools useable through the GUI again after the adaption. As the documentation of the Talend Open Studio source of IDRT is very limited (spare use of comments in the source code for example), a comprehensive knowledge of ETL with Talend Open Studio and—due to the complexity of the IDRT source—a considerable amount of time to understand the source is needed to perform adaptions. Especially when errors occur during the loading process, the IDRT only comes up with simple Java stack traces most of the time, which is probably not sufficient for nontechnicians who try to solve possible problems with input data. For this debugging process, further knowledge is recommended as well.

### 4.5. Limitations

We used data from TBase, which is a proprietary system and defines its own data schema. Nevertheless, relational databases and structures like EAV for medication data or laboratory values in source data are common use and transferable to other use cases as well.

The methodological mapping of the TBase data schema was made to the best of our knowledge and expertise. However, better solutions than ours cannot be precluded.

We analyzed four questions of nephrologists on cohort identification. Since this is a relatively small amount, it was not examined if the questions can be answered as they are; instead, we decomposed them into several inclusion and exclusion criteria (cf. Evaluation). By doing so, we classified the questions into eight different main criteria. The TBase database experts confirmed that these are the main criteria they have to deal with when they receive questions from nephrologists.

Moreover, we exclusively concentrated on the feasibility of the data mapping so that it was not evaluated if the queries on the i2b2 web client yield the same result as the original cohort identification queries on TBase. However, we logged any error messages of the IDRT during the loading process and finally performed a comparison of counts of facts for each entity on both databases to make sure that all datasets have been loaded correctly. Furthermore, the algorithm for mapping the relations between the facts has been described to the TBASE expert and was confirmed to be reasonable. Given the constraints of our setting, this kind of face validity had to suffice.

When integrating data with the IDRT, it creates a basic ontology in i2b2 that relates to the structure of the input data. For that reason, the IDRT comes with a graphical ontology editor. In this project, we did not describe the modeling of the ontology as we focused on the mapping and loading procedure. For an ideal solution in practice, it is recommended to model a proper ontology and also take medical terminologies into account that can be created with the IDRT ontology editor as well. As far as we know, however, the terminologies in the ontology editor are not updated anymore. The ICD10 terminology for example is only available up to 2015 so that any changes (new or substituted codes since then) in the terminology after 2015 are not available. This would lead to missing mappings of facts that comprise codes after 2015.

## 5. Conclusion

We showed how to extract, transfer, and load a complex relational database schema into the i2b2 star schema by the help of the IDRT. Afterwards, we determined whether common research questions can be answered by the help of the i2b2 web client or, alternatively, in the i2b2 database by the use of SQL. It turned out that our way of modeling the source data in i2b2 and the range of the i2b2 GUI functions allow for the latest research questions to be answered without the use of custom queries in the database.

Moreover, the IDRT accepts many different input sources and—according to our literature research—appears to be the only integration tool for i2b2 which accepts an input format that does not need to be provided in a proprietary format. Furthermore, we observed that the IDRT works well with a smaller amount of input data but encounters resource difficulties with a growing amount of input data. This is why we recommend the IDRT either for the import of smaller studies (up to 100 megabytes per file maximum) or for its use by nontechnicians, who can easily handle it because of its GUI-based front end. When it comes to the handling of comprehensive hospital databases with a huge amount of input data and data structures that are not originally supported by the IDRT (designated as tables of type 1/4/5 in the manuscript), a proprietary ETL solution is recommended instead.

## Figures and Tables

**Table 1:** Showing criteria to study mapping and presence of issues in the IDRT or i2b2.

Criterion	Study 1	Study 2	Study 3	Study 4	Issues with IDRT	Issues with i2b2
1	X				Y	N
2	X	X		X	N	N
3			X	X	N	N
4		X		X	N	N
5			X	X	N	N
6		X			Y	N
7a	X	X	X		N	N
7b				2X	N	N (as of i2b2 v1.7.00)

## Data Availability

The Integrated Data Repository Toolkit which has been analyzed during the current study is available in the repository of the University of Goettingen and on github, https://community.i2b2.org/wiki/display/IDRT/310.+Download.

## Abbreviations

BMWi: German Federal Ministry of Economic Affairs and Energy
CKD: Chronic kidney disease
DBMS: Database management system
EAV: Entity-attribute-value
ELT: Extract, load, and transform
ESA: Erythropoiesis-stimulating agents
ETL: Extract, transform, and load
GUI: Graphical user interface
i2b2: Informatics for integrating biology and the bedside
IDRT: Integrated data repository toolkit
RDF: Resource description framework
UACR: Urine albumin-to-creatinine ratio.
